# An overview of the Japan Breast Cancer Research Group (JBCRG) activities

**DOI:** 10.1007/s12282-012-0420-8

**Published:** 2013-03-15

**Authors:** Shinji Ohno, Katsumasa Kuroi, Masakazu Toi

**Affiliations:** 1 Department of Breast Oncology, National Kyushu Cancer Center, Notame 3-1-1 Minami-ku, Fukuoka, 811-1395 Japan; 2 Department of Surgery, Tokyo Metropolitan Cancer and Infectious Diseases Center Komagome Hospital, Tokyo, Japan; 3 Department of Surgery (Breast Surgery), Graduate School of Medicine, Kyoto University, Kyoto, Japan

**Keywords:** Clinical trials, Clinical research, Preoperative systemic therapy, Breast cancer

## Abstract

The purpose of this article is to describe the current status and future perspectives of the Japan Breast Cancer Research Group (JBCRG). The JBCRG was organized in 2002, with the following purpose: to plan and promote clinical trials and basic research in breast cancer domestically and multilaterally; to conduct research and surveys on domestic and foreign information on medical care for breast cancer and to diffuse and highlight such information; to improve and promote clinical technologies for breast cancer; to act as an intermediary to liaise and strengthen alliances with affiliated organizations; and, to contribute to the public welfare by improving outcomes in breast cancer. The clinical trials are led by doctors/investigators in the JBCRG. And the purpose is to establish standard treatment for patients and provide substantial evidence. The JBCRG implements international collaboration in some researches/studies. As of January 2012, fourteen trials have been closed and nine are open to recruitment.

## Introduction

The incidence of breast cancer in Japan has increased yearly; thus, more attention has been given to breast cancer treatment, among all cancers. In order to save as many breast cancer patients as possible, and to improve their quality of life (QOL), new diagnostic methods, treatments, and prophylaxes for breast cancer should be developed.

The JBCRG shall carry out the following, to serve the aforementioned purpose:

1. Basic and clinical research
2. Collection, analysis, and publication of information
3. Mutual exchange of information
4. Ordinary/extraordinary general meetings
5. Any other affairs required to accomplish the purpose of the JBCRG

The JBCRG has conducted mainly phase II trials to give answers to clinical questions, and now is planning to start phase III ones to achieve clinical approval of new standard therapies. The JBCRG is soliciting donations from organizations and individuals who wish to support its activities. The JBCRG usually manages data quality by central monitoring at data centers including the JBCRG Data Center, which is located in the Kyoto Technoscience Center, Kyoto; however, in some studies such as the SOLE trial, the JBCRG conducted site visits for source document verification.

As of January 2012, 243 doctors from 154 institutes are registered as JBCRG members who are specialists from the breast cancer treating hospitals around Japan. Also, the JBCRG is a member of the Breast International Group (BIG), which is an international breast cancer research group. Tables 1 and 2 summarize the closed and ongoing clinical trials, respectively.

**Table 1:** JBCRG trials closed/in follow-up

Trial	Design	No. of pts	Primary endpoints	Regimen	Enrollment start date
Neoadjuvant setting					
1	Phase II	202	Clinical response, safety	FEC100 q3w×4 → Doc75 q3w×4	Jun 02
2	Phase II	31	Clinical response, safety	FEC100 q3w×4 → Doc100 q3w×4	Aug 04
2′	Validation	19	Clinical response, histological effects, safety	FEC100 q3w×4 → Doc100 q3w×4	Dec 05
3	Phase II	130	Histological effects, safety	Doc75 q3w×4 → FEC100 q3w×4	Oct 05
5	Phase II	33	Response rates	Doc75 q3w×4 → letrozole 12 (−18) w	Sep 07
6	Phase II	40	Response rates	Letrozole 12 (−18) w	Sep 07
7	Phase II	40	Response rates	Letrozole + cyclophosphamide 24 w	Oct 07
10	Randomized phase II	180	Pathological CR rate	(1) FEC×4 → TCH×4, (2) TCH×4 → FEC×4, (3) TCH×6	Jun 09
13	Phase II	40	Pathological CR rate	Metronomic PCX 4 → FEC×4	Jan 10
Postoperative setting					
4 (CREATE-X)	Phase III	900	Disease-free survival	Any preoperative systemic therapy ± capecitabine	Feb 07
SOLE with BIG	Phase III	4,800	Disease-free survival	Intermittent or continuous letrozole	Apr 10
8 ALTTO	Phase III	140	Disease-free survival	Lapatinib and/or trastuzumab	Jul 07
Metastatic setting					
M01	Phase I	6	MTD, DLT, RD	CPT11 + S1	Jul 06
M01	Phase II	37	Response rates, clinical efficacy	CPT11 + S1	Jul 06
M02	Phase II	50	Response rates	Letrozole	Nov 06
Cohort study					
C01	Cohort	1,500	Disease-free survival	Trastuzumab	Sep 07

Data correct as of 31 March 2012

*CR* complete response, *MTD* maximum tolerated dose,* DLT* dose limiting toxicity,* RD* recommended dose,* FEC* 5-fluorouracil + epirubicin + cyclophosphamide,* Doc* docetaxel,* TCH* docetaxel + cyclophosphamide + trastuzumab, *PCX* paclitaxel + cyclophosphamide + capecitabine

**Table 2:** JBCRG trials open to recruitment

Trial	Design	No. of pts	Primary endpoints	Regimen	Enrollment start date
Neoadjuvant setting					
9	Randomized phase II	195	Histological response	TC×6, FEC×3 → TC×3,TC×3 → FEC×3	Sep 09
11CPA	Phase II	55	Response rates	Letrozole ± low dose cyclophosphamide	Oct 10
11TC	Phase II	60	Clinical response	Exemestane 12w or exemestane 12w+TC×4	Oct 10
Postoperative setting					
15	Phase II	30	Pharmacokinetics	Toremifene	Mar 09
SUPREMO with IBCSG	Phase III	3,700	Overall survival	Chest wall radiation	Jul 09
Metastatic setting					
12	Phase II	200	CYP2D6 and pharmacokinetics	Tamoxifen and toremifene	Jan 10
Cohort study					
C02	Cohort	100	Progression-free survival	Trastuzumab	Jul 09

Data correct as of 31 March 2012

*TC *docetaxel + cyclophosphamide, *FEC* 5-fluorouracil + epirubicin + cyclophosphamide

### Neoadjuvant pharmacotherapy

The first clinical trial conducted by the JBCRG was JBCRG-01, a phase II trial of preoperative systemic therapy (PST) using fluorouracil, epirubicin, and cyclophosphamide (FEC) followed by docetaxel (Doc) in patients with primary operable breast cancer [1–3]. Subsequently, JBCRG-02 study was conducted using FEC followed by Doc100 to investigate the safety and feasibility of 100 mg/m^2^ Doc as PST. JBCRG-03 was a study to clarify the most effective sequence of FEC and Doc75 [4]. From the results of these studies, we defined new criteria of pathological response to PST, quasi pathological complete response (QpCR), total or near total disappearance of the invasive tumor in the removed breast. QpCR following preoperative chemotherapy predicts favorable disease-free survival (DFS). HER2 overexpression and clinical response to FEC predict QpCR [5, 6].

#### JBCRG-01

JBCRG-01 was started in 2002 [1–3]. This multicenter phase II study examined the impact of pathological effect on survival after preoperative chemotherapy in Japanese women with early-stage breast cancer (ESBC). Prior to surgery, patients received four cycles of FEC (fluorouracil 500 mg/m^2^, epirubicin 100 mg/m^2^, cyclophosphamide 500 mg/m^2^ q3w) followed by four cycles of docetaxel (75 mg/m^2^ q3w). The primary endpoint was 3-year DFS stratified by the absence or presence of QpCR (absence of invasive tumor or only focal residual tumor cells). Secondary endpoints were predictors for QpCR, clinical response, breast conservation rate, and safety. Between June 2002 and June 2004, 202 women were enrolled. Among 191 assessable patients, 25 % achieved QpCR. With 40 months median follow-up, 3-year DFS was estimated at 91 % for all patients. The 3-year DFS for patients with QpCR was 98 vs. 89 % for those without QpCR (hazard ratio 0.38 [95 % confidence interval 0.09–0.84], *P* = 0.0134). HER2 status and response to FEC were independent predictors of QpCR. The overall clinical response rate was 75 %; 85 % of patients achieved breast conservation. Grade 3/4 neutropenia was the most common adverse event, observed in 44 and 35 % of patients during FEC and docetaxel treatment, respectively. Treatment-related side effects were manageable; there were no treatment-related fatalities.

#### JBCRG-02

The JBCRG-02 study was conducted to evaluate the safety and clinical and histologic effects of primary systemic chemotherapy using FEC followed by docetaxel in primary breast cancer. The primary endpoints were safety and clinical and histologic effects. Secondary endpoints were breast-conserving rate and DFS. Fluorouracil 500 mg/m^2^, epirubicin 100 mg/m^2^, and cyclophosphamide 500 mg/m^2^, q3w ×4 cycles, were followed by docetaxel 100 mg/m^2^, q3w ×4 cycles, as primary systemic chemotherapy. Among patients receiving this regimen, 19.5 % experienced a pathological complete response and 9.7 % had a near pathological complete response, resulting in a QpCR of 29.2 %.

#### JBCRG-03

JBCRG-03 was a multicenter, open-label, single-arm, phase II study assessing the efficacy of a neoadjuvant chemotherapy with docetaxel (75 mg/m^2^ q3w) followed by 5-fluorouracil 500 mg/m^2^, epirubicin 100 mg/m^2^, and cyclophosphamide 500 mg/m^2^ q3w in patients with ESBC [4]. The primary endpoint was the pathological complete response (pCR) rate defined for the breast alone, assessed by a central review committee. Secondary endpoints included clinical response and safety. Of the 132 patients assessable for pathologic response, 23 % experienced a pCR and 6 % had a near pathological complete response (few remaining cancer cells), resulting in a QpCR of 29 %. Clinical response rate following the initial docetaxel regimen was 64 %. The overall clinical response rate was 79 %. Breast-conserving surgery was performed in 79 % of patients. More patients with triple-negative disease experienced a pCR (14/29, 48 %) versus those with other molecular subtypes. The safety profile was acceptable.

### Oncotype DX

The 21-gene signature has been intensively studied and incorporated into major guidelines for treatment decision in early breast cancer. However, it remains to be examined whether this system is applicable to Asian populations.

#### Retrospective analysis

Toi et al. [7] were the first report to show that the 21-gene signature has value in providing prognostic information in Asian populations with estrogen receptor (ER)-positive, lymph node (LN)-negative breast cancer. A total of 325 tumor tissues were collected from ER-positive primary breast cancer patients who had undergone surgery and were treated with tamoxifen between 1992 and 1998. The tissues were analyzed for the 21-gene signature, and the patients were classified into groups of low, intermediate, or high risk on the basis of the recurrence score. A total of 280 patients were eligible, with adequate reverse transcription polymerase chain reaction profiles for the recurrence score. Of those, 200 and 80 patients had LN-negative and LN-positive disease, respectively. The proportions of LN-negative patients categorized as being at low, intermediate, or high risk were 48, 20, and 33 %, respectively. In LN-negative patients, the Kaplan–Meier estimates of the distant recurrence rate at 10 years were 3.3 % (95 % CI 1.1–10.0 %), 0 %, and 24.8 % (95 % CI 15.7–37.8 %) for those in the low-risk, intermediate-risk, and high-risk groups, respectively. The risk of distant recurrence in the low-risk group was significantly lower than that in the high-risk group when the entire Kaplan–Meier plots were compared (*P* < 0.001, log-rank test). There was a significant difference for overall survival between the low-risk and the high-risk groups (*P* = 0.008, log-rank test).

### Economic evaluation

#### JBCRG-TR03

This study evaluates the cost-effectiveness of two scenarios designed to include the assay into Japan’s social health insurance benefit package: one for LN−, ER+, ESBC and another for LN±, ER+, ESBC [8]. An economic decision tree and Markov model under Japan’s health system from the societal perspective is constructed with new evidence from the Japanese validation study. Incremental cost-effectiveness ratios are estimated as ¥384,828 (US$3,848) per quality-adjusted life year (QALY) for the LN− scenario and ¥568,533 (US$5,685) per QALY for the LN± scenario. Both estimates are not more than the suggested social willingness-to-pay for one QALY gain from an innovative medical intervention in Japan, ¥5,000,000/QALY (US$50,000/QALY). Sensitivity analyses show that this result is plausibly robust, because the incremental cost effectiveness ratios (ICERs) do not exceed the threshold despite various changes of assumptions made and values employed. Therefore, the inclusion of the assay in Japan’s social health insurance benefit package for not only LN− diseases but also LN+ diseases is cost-effective. Such a decision can be justifiable as an efficient use of finite resources for health care.

### Toxicity

Steroids and H(2) blockers are commonly used as supportive care for taxane-containing chemotherapy, but they also affect docetaxel’s primary metabolizer, cytochrome P(450) 3A4. Kawaguchi et al. [9] performed a retrospective observational study to better understand the effects of these compounds on docetaxel-induced skin toxicities, specifically hand-foot syndrome (HFS) and facial erythema (FE), a relationship that is currently poorly understood. Member institutions of the JBCRG were invited to complete a questionnaire on the occurrence of grade 2 or higher HFS and FE among patients treated between April 2007 and March 2008 with docetaxel as an adjuvant or neoadjuvant chemotherapeutic treatment for breast cancer. We obtained data for 993 patients from 20 institutions. Twenty percent received H(2) blockers, and all patients received dexamethasone. Univariate and multivariate analyses revealed that H(2) blockers are associated with a significantly higher incidence of both HFS and FE. The incidence of FE was significantly higher for the docetaxel + cyclophosphamide (TC) regimen than for non-TC regimens combined. Dexamethasone usage did not affect the incidence of either HFS or FE. In conclusion, the use of H(2) blockers as premedication in breast cancer patients receiving docetaxel significantly increases the risk of both HFS and FE.

### International study

The JBCRG is a member of the international breast cancer research group BIG. The JBCRG has joined in with several international clinical studies.

#### JBCRG-04 (CREATE-X)

This study aims to investigate the efficacy and safety of capecitabine, as a postoperative adjuvant chemotherapy, for breast cancer patients who were pathologically demonstrated to have residual cancer cells after the preoperative chemotherapy. In addition, the cost-effectiveness of capecitabine is to be investigated. The primary objective is DFS and secondary ones are overall survival, safety, and cost-effectiveness. Eligible patients had stage I-IIIB at the first diagnosis (curable breast cancer) and were non-pCR after preoperative chemotherapy including at least two cycles anthracycline agents; that is, they were confirmed pathologically by surgical and/or histological tests to have residual cancer cells. The patients had also been confirmed to be HER2 negative.

#### JBCRG-08 (ALTTO)

JBCRG-08 was a randomized, multicenter, open-label, phase III study of adjuvant lapatinib, trastuzumab, their sequence, and their combination in patients with HER2/ErbB2-positive primary breast cancer (BIG 2-06/N063D/EGF 106708.). The objective of this study was to compare DFS in patients with HER2 overexpressing and/or amplified breast cancer randomized to trastuzumab for 1 year versus lapatinib for 1 year versus trastuzumab (12 weeks) followed by a 6-week treatment-free interval followed by lapatinib (34 weeks) versus trastuzumab in combination with lapatinib for 1 year. Endpoints were DFS, overall survival (OS), time to recurrence (TTR), time to distant recurrence (TTDR), safety and tolerability, cumulative incidence of brain metastases as the first site of breast cancer recurrence, presence or absence of cMyc gene amplification, expression levels of PTEN, and presence or absence of p95 HER2 domain. Trial periods were between July 2007 and February 2011 (registration, 2 years; follow-up study, 5 years). Target sample size was 140 from 15 institutions.

#### SOLE trial

SOLE trial is a phase III trial evaluating the role of continuous letrozole versus intermittent letrozole following 4–6 years of prior adjuvant endocrine therapy for postmenopausal women with hormone receptor-positive, node-positive ESBC. The JBCRG is collaborating with the International Breast Cancer Study Group (IBCSG) on this trial. A total of 4,800 patients are expected to be enrolled in this study. The primary endpoint is DFS, and secondary ones are OS, distant DFS, breast cancer-free interval, sites of first failure, second (non-breast) malignancies, deaths without prior cancer events, and adverse events.

#### SUPREMO trial

The SUPREMO trial is a randomized phase III trial assessing the role of chest wall irradiation in women with intermediate-risk breast cancer following mastectomy conducted by BIG. Postoperative radiotherapy is routinely given to patients at higher risk of recurrence with 4 or more LNs or large tumor(s). In patients with less than 4 LNs under the armpit involved by cancer or with no LNs involved but other features of the cancer that increase the risk of recurrence, it is not clear whether postoperative radiotherapy is needed. Eligibility criteria are a postoperative breast cancer patient who has had a mastectomy, and who has an intermediate risk of the cancer returning. An intermediate risk is diagnosed when there are less than 4 LNs under the armpit involved by cancer or there are no LNs involved, but there are other features of the cancer that mean it is more likely to come back. The trial will involve 1,600 women.

## Conclusion

The JBCRG was founded in order to perform good-quality multicenter studies, and related clinical trials in close liaison with research institutions in other countries and regions, as well as in Japan. The JBCRG has performed a variety of studies, including primary pharmacotherapy, pharmacotherapy for recurrent breast cancer, clinical trials on postoperative pharmacotherapy, prediction of prognosis in hormone receptor-positive breast cancer, and prediction of the effect of chemotherapeutic drugs. The JBCRG has reported a number of outcomes to academic societies and in journals, and has obtained a good reputation. The incidence of breast cancer in Japan has increased yearly; thus, more attention has been given to breast cancer treatment, among all cancers. In order to save as many breast cancer patients as possible, and to improve their QOL, we will develop new diagnostic methods, treatments, and prophylaxes for breast cancer.
